# The metabolic endotoxemia and gut microbiota: research trajectories and hot trends across the centuries (1999–2024)

**DOI:** 10.3389/fmicb.2025.1634803

**Published:** 2025-09-05

**Authors:** Enlin Jian, Mengping Wang, Zhen Zhang, Yiwei Heng, Chengwei Zhang, Qing Chen, Xiaoping Yu, Yanfeng Zhu, Peiling Cai

**Affiliations:** ^1^School of Preclinical Medicine, Chengdu University, Chengdu, Sichuan, China; ^2^Clinical Medical College & Affiliated Hospital of Chengdu University, Chengdu, Sichuan, China; ^3^School of Public Health, Chengdu Medical College, Chengdu, Sichuan, China

**Keywords:** metabolic endotoxemia, gut microbiota, bibliometric analysis, hotspots, research trend

## Abstract

**Background:**

Metabolic endotoxemia, characterized by chronic low-grade elevation of circulating bacterial endotoxins such as lipopolysaccharides derived from Gram-negative bacteria, has emerged as a central focus in metabolic disease research. Notably, emerging evidence highlights the pivotal role of gut microbiota in driving this condition, though critical knowledge gaps persist regarding the precise mechanisms mediated by gut microbiota-derived metabolites and their specific signaling pathways, as well as the development of individualized intervention strategies. To systematically map the evolving research landscape, this review employs bibliometric analysis to identify emerging trends and patterns in understanding the interplay between metabolic endotoxemia and gut microbial communities.

**Materials and methods:**

This study employed a comprehensive bibliometric analysis of scholarly literature indexed in the Web of Science Core Collection (1900–2024) to investigate research patterns in metabolic endotoxemia and gut microbiota. Through multidimensional examination using VOSviewer, CiteSpace, the bibliometrix R package, and the online bibliometric analysis platform (https://bibliometric.com/), we systematically mapped research priorities, collaborative networks, and evolving frontiers in this interdisciplinary field. The methodology incorporated advanced visualization techniques and quantitative assessments to identify thematic clusters, institutional contributions, and knowledge diffusion pathways within the extant literature corpus.

**Results:**

This comprehensive global analysis, encompassing 1,585 relevant studies, demonstrates compelling evidence that China and USA collectively contribute over 50% of scientific output in this specialized research domain. Notably, academic investigations into metabolic endotoxemia and gut microbiota continue to maintain substantial research momentum. Current scholarly discourse prominently features five key thematic clusters: gut dysbiosis, intestinal barrier, risk, gut-liver axis, and short-chain fatty acids.

**Conclusion:**

This in-depth review systematically examines the evolving research landscape and emerging investigative priorities in metabolic endotoxemia and gut microbiota. It equips researchers with a comprehensive overview of key contributors in these specialized fields, including leading nations, institutions, academic journals, and potential collaborative networks. The analysis further delineates cutting-edge research frontiers and proposes strategic directions for future investigation, thereby serving as an invaluable resource for scholars navigating the complex interplay between metabolic endotoxemia and gut microbiota in the intestinal ecosystem.

## 1 Introduction

Metabolic endotoxemia has emerged as a pivotal concept in contemporary metabolic disease research, characterized by chronically elevated low-level circulating endotoxins, particularly lipopolysaccharides (LPS) derived from Gram-negative bacterial cell walls (Cani et al., 2007). The World Health Organization recognizes endotoxin-induced chronic inflammation as a key mediator in the pathogenesis of noncommunicable diseases, which account for 74% of global deaths annually (WHO, 2024). Under physiological conditions, these endotoxins are effectively contained by the intestinal barrier (Cani et al., 2007; Ghoshal et al., 2009). However, compromised intestinal permeability enables LPS translocation into the portal circulation, initiating systemic low-grade inflammation (Blanchard et al., 2017). This condition demonstrates strong pathophysiological associations with obesity (Liang et al., 2013), insulin resistance (Jin et al., 2014), type 2 diabetes mellitus (Pussinen et al., 2011), and non-alcoholic fatty liver disease (Jin et al., 2014). Mechanistically, dietary fat intake (Cani et al., 2007; Netto Candido et al., 2018) and gut microbiota dysbiosis (Devkota et al., 2012) significantly elevate plasma LPS concentrations. Subsequent activation of the Toll-like receptor 4 (TLR4) signaling cascade drives the production of pro-inflammatory cytokines (e.g., TNF-α, IL-6). Mechanistic studies reveal that dietary fat intake (Cani et al., 2007; Netto Candido et al., 2018) and gut microbiota dysbiosis (Devkota et al., 2012) significantly increase plasma LPS concentrations, which subsequently activate the TLR4 signaling cascade. This activation drives the production of pro-inflammatory cytokines (e.g., TNF-α, IL-6) that disrupt insulin signaling pathways, exacerbate adipose tissue inflammation, and promote hepatic lipid deposition (Bannerman and Goldblum, 1999; Asehnoune et al., 2004; Ryan et al., 2004; Cani et al., 2007; Lu et al., 2008). It is important to highlight that the pathological effects of metabolic endotoxemia are dose-dependent, and prolonged accumulation may result in chronic metabolic disorders (Mills et al., 1995). This highlights its important role as a hidden intrinsic driving force in the development of metabolic syndrome.

The human gut microbiota, commonly referred to as the gut microbiota, consists of ~100 trillion microbes and contains a wide variety of microorganisms including bacteria, archaea, viruses, and eukaryotes (Ley et al., 2006). The gut microbiota plays a fundamental role in host physiology (Schroeder and Bäckhed, 2016), orchestrating nutrient metabolism (Cani et al., 2008), immune regulation (Belkaid and Harrison, 2017), and preservation of intestinal barrier integrity (Nicholson et al., 2012). This multilayered barrier, composed of mucus secretions, epithelial cells, and immune components, forms the vital protective boundary that maintains separation between luminal microorganisms and host tissues (Turner, 2009). Under physiological conditions, tight junction proteins such as occludin and zonula occludens-1 (ZO-1) maintain selective paracellular permeability, effectively preventing microbial translocation while permitting regulated nutrient absorption (Turner, 2009). However, dysbiosis (microbial community imbalance) disrupts this homeostatic equilibrium. Pathological alterations in microbial composition not only compromise intestinal tight junctions, precipitating hyperpermeability (Lynch and Pedersen, 2016), but also dysregulate microbial-derived metabolites—characterized by elevated secondary bile acids and diminished butyrate synthesis (Wahlström et al., 2016; Canfora et al., 2019). These dual mechanisms of barrier dysfunction and metabolic perturbation establish gut dysbiosis as a critical environmental determinant in the pathogenesis of metabolic disorders.

A reciprocal relationship exists between imbalanced gut bacteria and elevated blood endotoxin levels, creating a self-sustaining loop where compromised intestinal barrier function, systemic inflammation, and metabolic abnormalities mutually reinforce each other (Cani et al., 2007; Hasain et al., 2020). Imbalance in the gut microbiota disrupts the intestinal barrier by downregulating tight junction proteins (e.g., occludin and ZO-1), promoting endotoxin translocation (Bian et al., 2022). Mouse experiments revealed that a 4-week high-fat diet increased plasma LPS levels by 2-3 times, accompanied by expansion of Proteobacteria (Cani et al., 2007; Bian et al., 2022). Endotoxins activate the TLR4/nuclear factor kappa B (NF-κB) pathway, triggering systemic inflammation and exacerbating insulin resistance and lipid metabolism disorders (Ding et al., 2023). Clinical studies found enrichment of LPS-producing Enterobacteriaceae in the gut of type 2 diabetes patients, positively correlated with HbA1c levels (Qin et al., 2012). Importantly, disturbances in metabolic regulation (e.g., hyperglycemia) paradoxically stimulate the overgrowth of Enterobacteriaceae species, whereas elevated free fatty acid levels simultaneously impair the establishment of beneficial Bifidobacterium communities, thereby creating a self-reinforcing pathological cycle (Cani et al., 2008; Fuke et al., 2019). These mechanistic insights not only establish the gut microbiota-endotoxin axis as a central mediator of metabolic disorders but also pioneer novel microbiome-targeted precision therapeutic approaches.

In recent years, reviews on the interaction between metabolic endotoxemia and gut microbiota have systematically elucidated the molecular mechanisms and clinical significance of these phenomena in disease pathogenesis. Cani et al. (2007) highlighted that a high-fat diet alters the composition of the intestinal microbiota (e.g., increasing the proportion of gram-negative bacteria), leading to elevated LPS release, which triggers low-grade inflammation and insulin resistance. This process is closely associated with impaired intestinal barrier function. Vancamelbeke and Vermeire further explored the regulatory mechanisms of intestinal barrier permeability, emphasizing that dysregulation of tight junction proteins such as occludin and ZO-1 plays a crucial role in endotoxemia induced by microbial metabolites (Vancamelbeke and Vermeire, 2017). Recent studies have also focused on intervention strategies: Plovier et al. reviewed the potential of probiotics and prebiotics to restore microbiota balance and reduce circulating LPS levels (Plovier and Cani, 2017). Additionally, dietary fibers like butyrate can enhance intestinal barrier function through activation of GPR43/109A receptors (Kayama et al., 2020). Moreover, Sonnenburg and Backhed underscored the pivotal roles of the “gut-liver axis” and “gut-brain axis” in metabolic diseases, revealing that imbalances in the gut microbiota influence multi-organ inflammatory networks via the LPS-TLR4 signaling pathway (Sonnenburg and Bäckhed, 2016). Despite substantial progress in understanding the interactions between metabolic endotoxemia and gut microbiota, fundamental questions remain unresolved in this field. This context underscores the critical importance of applying bibliometric methodologies to systematically analyze core research directions, pivotal domains, and emerging trends through quantitative evaluation. As an essential branch of information science, bibliometric analysis employs mathematical and statistical methodologies to systematically assess academic literature, elucidating knowledge structures, characteristic attributes, and temporal evolution patterns (Cheng et al., 2022a; Wu et al., 2022; Su et al., 2023). Beyond revealing patterns of scholarly communication and knowledge dissemination, this approach leverages big data analytics to discern disciplinary trajectories and paradigm shifts (Cheng et al., 2022b; Zhu et al., 2022). Our study pioneers the integration of bibliometric techniques with visual analytics to conduct a comprehensive review of literature spanning January 1900 to December 2024. This study establishes the first systematic visualization of the metabolic endotoxemia-gut microbiota interface through bibliometric analysis. Employing comprehensive bibliometric methods from January 1900 to December 2024, we examine significant developments in this field to map current research domains, predominant themes, and hot trends.

## 2 Materials and methods

### 2.1 Database and search strategy

The Web of Science Core Collection (WoSCC) was selected as the primary data source for this investigation based on its comprehensive advantages in scholarly literature retrieval (Han et al., 2023). As a globally recognized authoritative citation database, WoSCC provides access to high-impact journals spanning multiple disciplines, offering broader subject coverage compared to PubMed's exclusive focus on biomedical literature (You et al., 2021). Particularly noteworthy is WoSCC's enhanced compatibility with CiteSpace software, which generates superior visualization outputs when constructing scientometric maps (Falagas et al., 2008). This operational advantage, combined with its established reliability as evidenced by extensive adoption in previous bibliometric studies and knowledge network analyses, solidifies WoSCC's position as an optimal single-source solution for quantitative research in science mapping (Gonzalez et al., 2020; Yan et al., 2020; Li et al., 2021).

**Figure 1:**
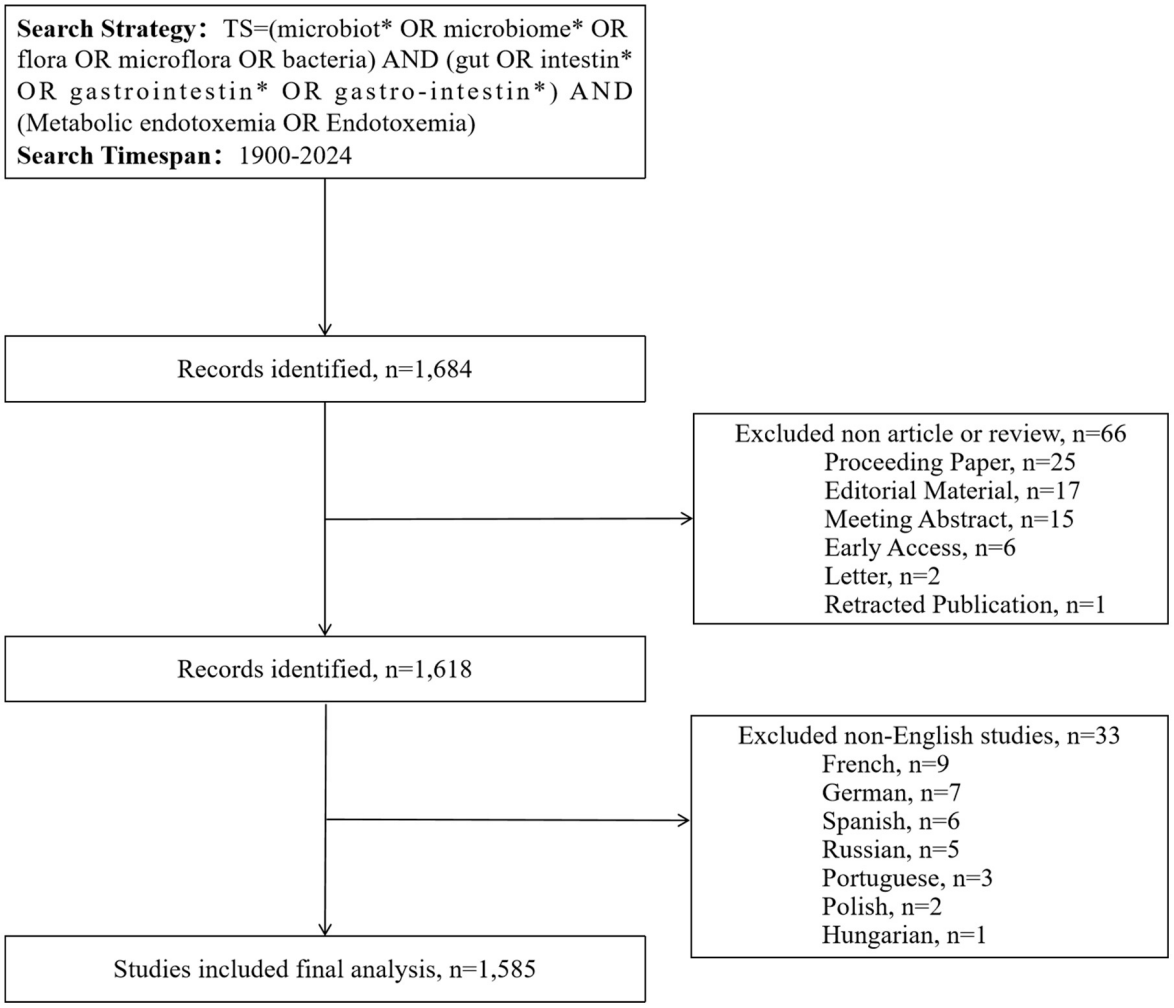
Flowcharts of the publication selection.

This study conducted a comprehensive literature search through the WoSCC database, covering publications from January 1, 1900 to December 31, 2024. Data collection was finalized on February 11, 2025, with the objective of systematically gathering core academic outputs within this timeframe. All retrieved records were preserved in text format to establish a structured database for subsequent analysis. The research approach employed a diverse set of essential keywords and their various pairings. The search strategy employed dual filters: linguistic restriction to English-language documents and categorical limitation to research articles and review papers. Due to the ongoing nature of 2025 literature indexing in database systems, the analytical sample was strictly confined to publications through 2024 to ensure the reliability of bibliometric outcomes. The methodological framework for literature screening and analysis is visually presented in Figure 1.

### 2.2 Proposed guidelines for research

This study adhered to the 2020 Preferred Reporting Items for Systematic Reviews and Meta-Analyses (PRISMA) statement reporting guidelines. The complete checklist is available in Supplementary Tables 1, 2. To further strengthen the methodological rigor, we also incorporated the Preferred Reporting Items for Bibliometric Analysis (PRIBA) checklist (Page et al., 2021; Koo and Lin, 2023), a specialized framework tailored to address the distinct reporting needs of bibliometric studies. Both checklists are included as Supplementary material (PRISMA in Supplementary Tables 1, 2 and PRIBA in Supplementary Table 3) to ensure adherence to the standards for both systematic reviews and bibliometric analyses. Adherence to these reporting guidelines ensures methodological transparency and provides readers with a robust framework for assessing the research design, implementation, and documentation.

### 2.3 Bibliometric analysis

This study conducted a systematic bibliometric investigation employing a comprehensive analytical framework that integrates VOSviewer (v.1.6.20), CiteSpace (v.6.3.R3 Advanced), R package “bibliometrix”, and online bibliometric platforms (https://bibliometric.com/). By leveraging the complementary strengths of VOSviewer and CiteSpace, we performed a sophisticated examination of core analytical modules including national co-occurrence networks, institutional collaboration networks, author co-citation networks, dual-map overlay of journals, document co-citation timeline mapping, and keyword burst detection mapping. Furthermore, through the combined application of R-based bibliometric packages and web-based analytical tools, we developed a multi-dimensional visual analytical framework encompassing annual publication trends, transnational collaboration patterns, author productivity distributions, and temporal evolution of journal contributions.

This research made use of anonymized data sourced solely from an open-access database, namely the WoSCC, and therefore did not require ethical review or approval from an institutional review board.

## 3 Results

### 3.1 Scientific output

**Figure 2:**
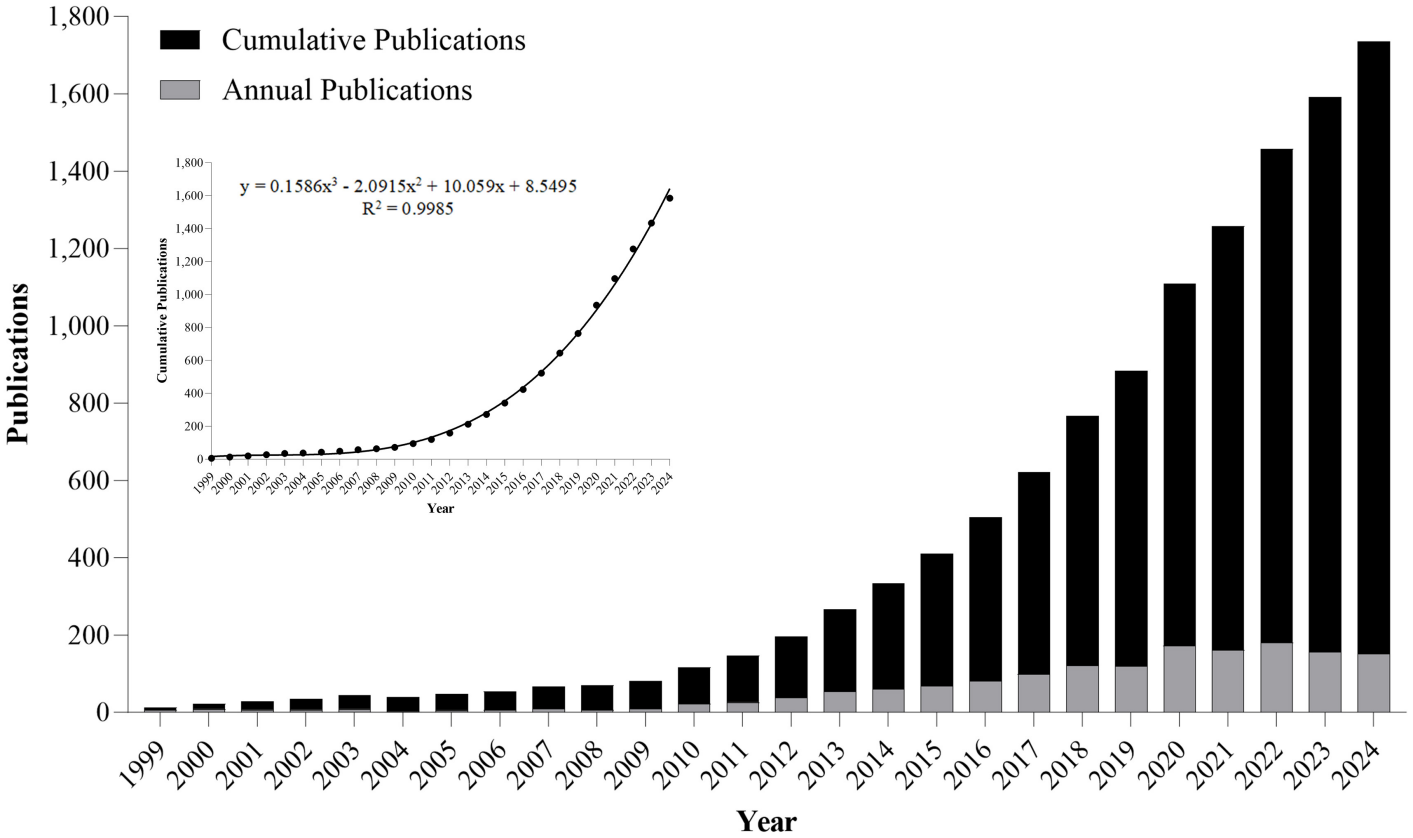
Annual and cumulative output of global metabolic endotoxemia and gut microbiota research publications from 1999 to 2024.

A systematic search across WoSCC (1900–2024) retrieved 1,585 peer-reviewed articles meeting predefined inclusion criteria (Figure 1). Our systematic analysis revealed three distinct research epochs: (1) Pre-1999 - no identifiable publications; (2) 1999-2009 - nascent phase with annual output ≤ 9 articles; (3) Post-2010 - exponential growth phase. Annual publication output in this field first exceeded the 100-article threshold in 2018, reaching its zenith at 180 papers in 2022 (Figure 2). Research output on metabolic endotoxemia and gut microbiota has maintained a 13.77% annual growth rate since 1999, with cumulative publications from 1999 to 2024 conforming to a cubic polynomial regression model (y = 0.1586x3 – 2.0915x^2^ + 10.059x + 8.5495; *R*^2^ = 0.9985) (Figure 2). Extrapolation of this trajectory indicates that cumulative publications will exceed 1,800 by 2025. Our results support the application of Bradford's law, a bibliometrics concept proposed by Brookes (1969).

### 3.2 Countries/regions

China and the United States of America (USA) were the leading contributors to research output on metabolic endotoxemia and gut microbiota (Table 1), collectively representing 50.67% of total publications among the top 10 countries/regions. When measured by H-index, the USA, China, and France ranked highest in research influence.

**Table 1:** Top 10 countries/regions in metabolic endotoxemia and gut microbiota, ranked by the number of publications.

**Rank**	**Countries/Regions**	**Publications**	**% of (1,585)**	**Citations**	**Average citations**	**H-index**
1	China	426	26.88%	21,054	49.42	75
2	USA	377	23.79%	32,688	86.71	93
3	Italy	96	6.06%	7,628	79.46	43
4	France	90	5.68%	16,508	183.42	46
5	Germany	76	4.79%	3,981	52.38	35
6	Spain	75	4.73%	4,966	66.21	37
7	Japan	73	4.61%	4,944	67.73	33
8	Canada	63	3.97%	3,519	55.86	30
9	Brazil	62	3.91%	2,868	46.26	28
10	England	59	3.72%	8,166	138.41	30

**Figure 3:**
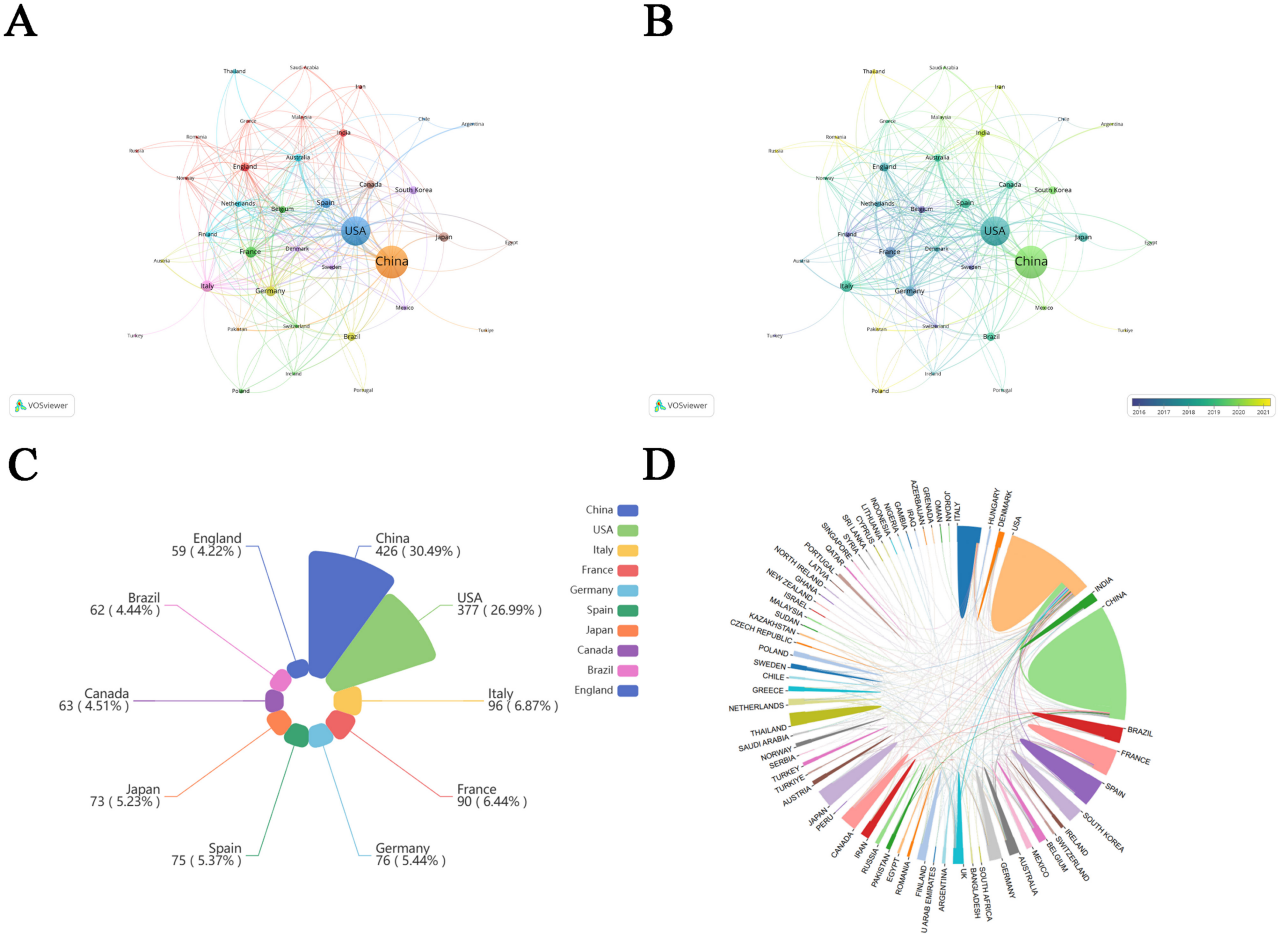
Country cooperation network map. **(A)** Visualization of journal publications in the field of metabolic endotoxemia and gut microbiota research. **(B)** Visual mapping of country co-author coverage using VOSviewer. The varying node colors in this visualization reflect the average appearance year (AAY) for each country, represented by the color gradient in the lower right corner. **(C)** Top 10 countries/regions in metabolic endotoxemia and gut microbiota using Nightingale rose plots. **(D)** The international collaboration among pertinent countries/regions.

Using VOSviewer software, we mapped international collaborative networks (Figures 3A, B), restricting analysis to countries/regions with over five qualifying publications to ensure meaningful visualization of academic partnerships. Among the top 10 countries/regions, China and USA accounted for more than 50%, indicating the importance and influence of China and USA in this field. Belgium, Switzerland, Sweden, and Finland were among the pioneering participants in the investigation of metabolic endotoxemia and gut microbiota. In recent years, Poland, Pakistan, Romania, Thailand, and Russia have also joined these efforts.

The top 10 countries/regions of contributing nations (Figure 3C) identified China and USA as the predominant contributors. Furthermore, network visualization (Figure 3D) revealed these two nations exhibited the strongest bilateral collaboration.

### 3.3 Institutions

**Figure 4:**
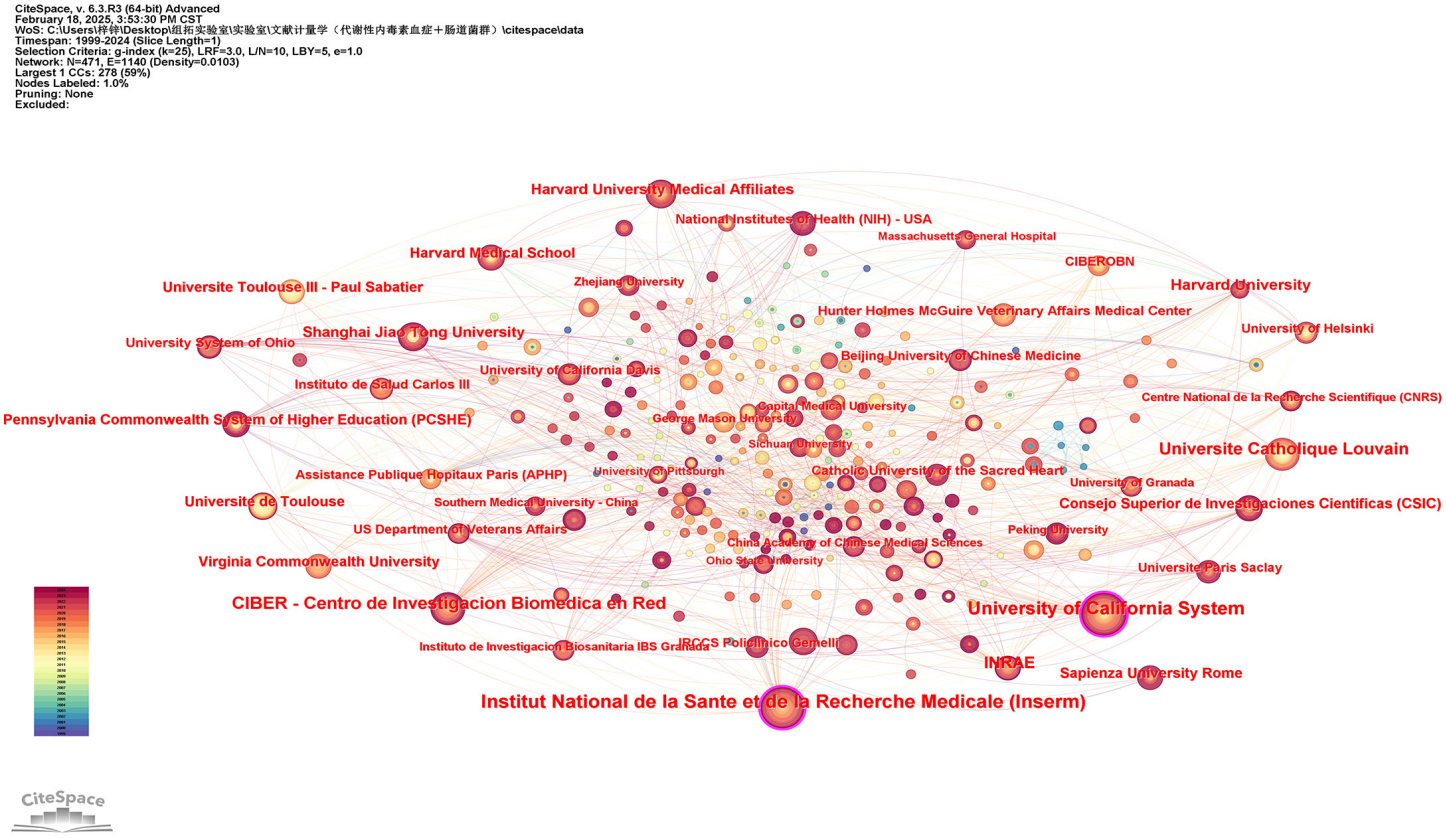
Institution network visualization produced using CiteSpace.

A total of over 2,000 institutions worldwide participated in global scientific research output, with CiteSpace detecting 471 notable contributors (Figure 4). As shown in Table 2, the ranking of top 10 institutions by research output features Institut National de la Santé et de la Recherche Médicale (Inserm) as the most productive entity with 64 studies, trailed by the University of California System (53 publications) and CIBER - Centro de Investigación Biomédica en Red (40 publications). When evaluating academic influence through the H-index metric, Inserm maintained leadership (H-index = 38), followed by Université Catholique Louvain (36) and the University of California System (32). Network analysis identified two core collaborative hubs: the University of California System demonstrated the strongest connective influence (Betweenness Centrality = 0.17), while Inserm showed significant interdisciplinary connectivity (BC = 0.12).

**Table 2:** Top 10 productive institutions in metabolic endotoxemia and gut microbiota, ranked by the number of publications.

**Rank**	**Institution**	**Country**	**Publications**	**% of (1,585)**	**Betweenness centrality**	**H-index**
1	Institut National de la Sante et de la Recherche Medicale (Inserm)	France	64	4.04%	0.12	38
2	University of California System	USA	53	3.34%	0.17	32
3	CIBER - Centro de Investigacion Biomedica en Red	Ethiopia	40	2.52%	0.08	27
4	Universite Catholique Louvain	Belgium	36	2.27%	0.04	36
5	INRAE	France	32	2.02%	0.08	20
6	Harvard University	USA	26	1.64%	0.09	18
7	Harvard University Medical Affiliates	USA	21	1.32%	0.02	17
8	Shanghai Jiao Tong University	China	21	1.32%	0.08	20
9	Universite de Toulouse	France	20	1.26%	0.01	20
10	Chulalongkorn University	Thailand	20	1.26%	0	15

### 3.4 Journals

Researchers have published the existing body of research in 593 scientific journals. Table 3 ranks the 20 most prominent publication venues in this field. At the forefront, the journal “Nutrients” stands tall with a substantial 61 articles (3.85%), boasting an impressive 3,299 citations and a robust link strength of 303. It is closely followed by “International Journal of Molecular Sciences” with 41 published articles (2.59%), “PLOS One” with 37 articles (2.33%), “Scientific Reports” with 36 articles (2.27%), and “Food & Function” with 27 articles (1.70%). Among these journals, “Gut Microbes” stood out as the publication with the most significant impact, achieving an impact factor (IF) (2023) of 12.2. As indicated in Table 4, the 10 most frequently co-cited journals each demonstrated substantial scholarly impact with co-citation frequencies exceeding 1,000 instances. The journal “Nature” emerged as the most prominent contributor with 3,237 co-citations, followed closely by “PLOS One” (2,754), “Diabetes” (2,074), “Gut” (2,214), and “Proceedings of the National Academy of Sciences of the USA of America” (2,165). Notably, “Nature” maintained its preeminent position not only in citation metrics but also in academic prestige, boasting an IF (2023) of 50.5 that underscores its exceptional influence within the research domain.

**Table 3:** The top 20 journals in terms of the number of publications relating to metabolic endotoxemia and gut microbiota.

**Rank**	**Source**	**Publications**	**% of (1,585)**	**Citations**	**Total link strength**	**IF (2023)**	**JCR**
1	Nutrients	61	3.85%	3,299	303	4.8	Q1
2	International Journal of Molecular Sciences	41	2.59%	887	180	4.9	Q1
3	PLOS One	37	2.33%	3,370	159	2.9	Q1
4	Scientific Reports	36	2.27%	3,117	177	3.8	Q1
5	Food & Function	27	1.70%	993	110	5.1	Q1
6	Molecular Nutrition & Food Research	25	1.58%	813	119	4.5	Q1
7	Frontiers in Microbiology	22	1.39%	1,483	111	4.0	Q2
8	Journal of Nutritional Biochemistry	20	1.26%	1,013	91	4.8	Q1
9	American Journal of Physiology-Gastrointestinal and Liver Physiology	19	1.20%	2,707	154	3.9	Q1
10	Gut Microbes	19	1.20%	1,778	135	12.2	Q1
11	Microorganisms	19	1.20%	358	73	4.1	Q2
12	Frontiers in Immunology	18	1.14%	1,068	96	5.7	Q1
13	World Journal of Gastroenterology	18	1.14%	1,332	72	4.3	Q1
14	Journal of Agricultural and Food Chemistry	16	1.01%	446	66	5.7	Q1
15	Frontiers in Cellular and Infection Microbiology	14	0.88%	581	49	4.6	Q1
16	Frontiers in Pharmacology	14	0.88%	387	45	4.4	Q1
17	Frontiers in Nutrition	13	0.82%	145	52	4.0	Q2
18	Journal of Nutrition	13	0.82%	548	46	3.7	Q2
19	Faseb Journal	11	0.69%	852	53	4.4	Q1
20	Journal of Functional Foods	11	0.69%	255	50	3.8	Q2

**Table 4:** The top 10 co-cited journals in terms of the number of publications relating to metabolic endotoxemia and gut microbiota.

**Rank**	**Source**	**Citations**	**Total link strength**	**IF (2023)**	**JCR**
1	Nature	3,237	175,651	50.5	Q1
2	PLOS One	2,754	142,539	2.9	Q1
3	Diabetes	2,223	107,797	6.2	Q1
4	Gut	2,214	115,699	23.1	Q1
5	Proceedings of the National Academy of Sciences of the United States of America	2,165	117,907	9.4	Q1
6	Gastroenterology	1,858	95,448	26.3	Q1
7	Hepatology	1,853	87,383	13.0	Q1
8	Nutrients	1,557	87,332	4.8	Q1
9	Science	1,459	86,284	44.8	Q1
10	Scientific Reports	1,421	79,923	3.8	Q1

**Figure 5:**
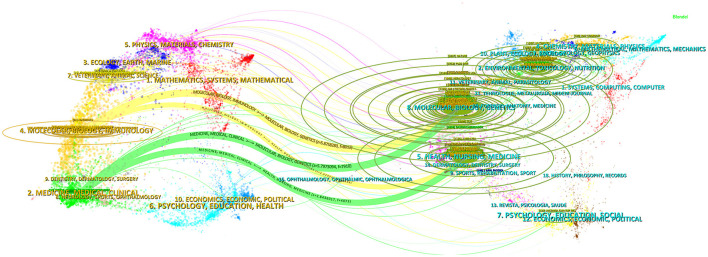
A dual-map overlay of journals that published literature in the research of metabolic endotoxemia and gut microbiota. The arc thickness quantitatively reflects connection intensity, where greater visual weight indicates stronger interdisciplinary relationships between subject areas.

The disciplinary affiliations of citing publications and their references primarily define scholarly connections. Through overlay mapping, CiteSpace reveals both domain-specific concentrations and interdisciplinary bridges within scholarly communication (Chen and Leydesdorff, 2013). In Figure 5, the left panel corresponds to disciplinary domains of citing literature, while the right panel represents those of cited references, with interconnecting arcs denoting citation flows. Among them, “Molecular, Biology, Immunology” is mainly based on the knowledge of such disciplines as “Molecular, Biology, Genetics” and “Health, Nursing, Medicine,” while “Medicine, Medical, Clinical” mainly refer to the knowledge of such fields as “Molecular, Biology, Genetics” and “Health, Nursing, Medicine.” At the same time, “Physics, Materials, Chemistry” and “Ecology, Earth, Marine” are also the focus areas of citing literature, while “Environmental, Toxicology, Nutrition” “Chemistry, Materials, Physics,” and “Psychology, Education, Social” are also important sources of knowledge for citing literature.

### 3.5 Authors

**Figure 6:**
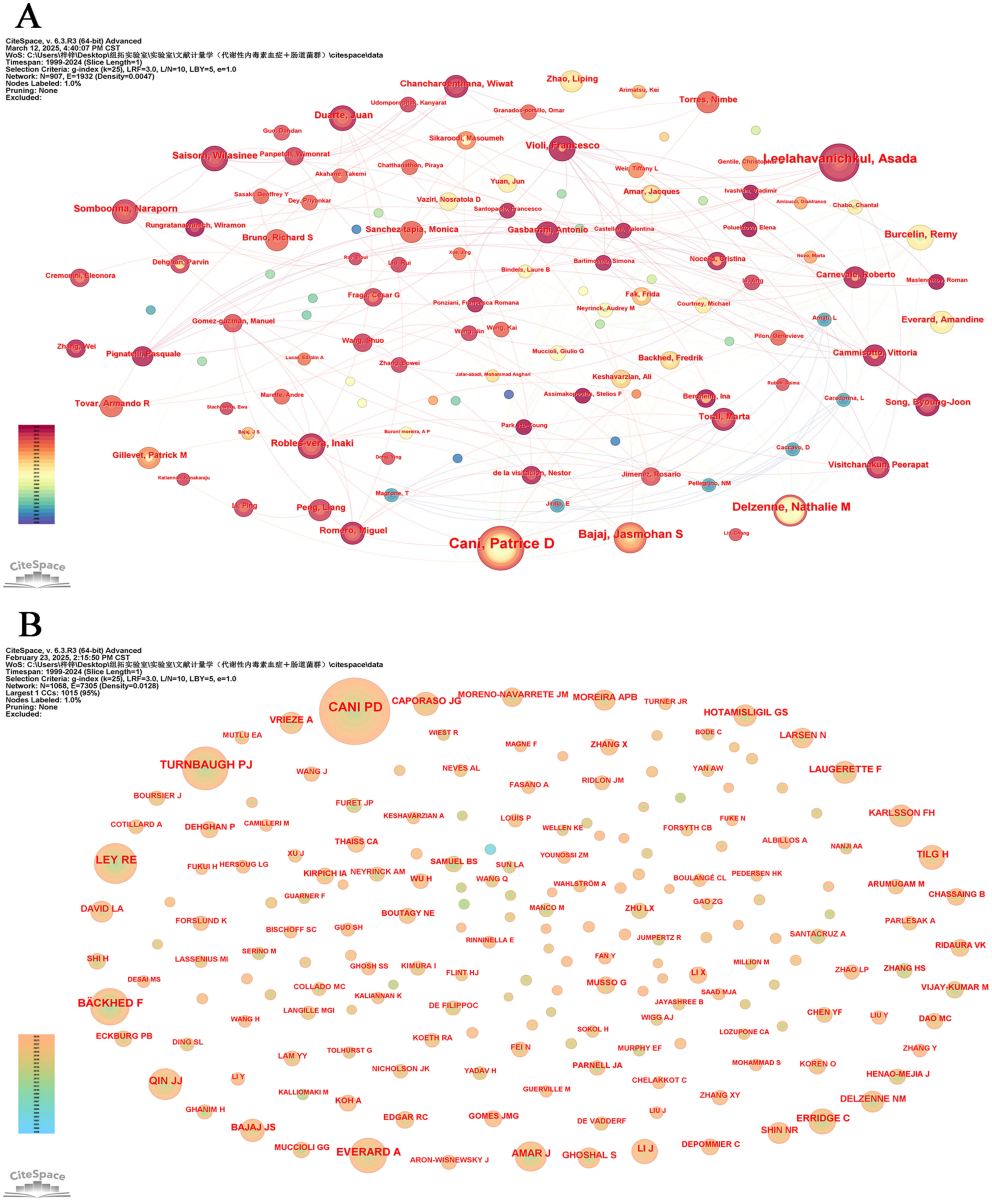
Author analysis. **(A)** Collaboration networks. Each node, distinguished by varying hues and sizes, represents an individual researcher. The size of a node is indicative of the number of publications, whereas the color signifies the year of publication. **(B)** Cited authors co-occurrence network.

In more than 25 years, from 1999 to 2024, 9,275 authors published an article exploring metabolic endotoxemia and gut microbiota. Table 5 presents the 15 most prolific scholars in this research domain along with their professional profiles. This cohort has collectively contributed 207 scholarly publications, representing 13.06% of total academic output in this field. Belgium researcher Cani, Patrice D. emerges as the core contributor, having produced 34 publications that specifically address metabolic endotoxemia in relation to gut microbiota dynamics. Following closely behind are his Thailand colleague Leelahavanichkul, Asada and Belgium expert Delzenne, Nathalie m., both of whom have established themselves as leading figures through sustained research efforts and consistent contributions to the discipline. The CiteSpace visualization portrays an intricate network of authors, who are actively researching the correlation between metabolic endotoxemia and gut microbiota (Figure 6A).

**Table 5:** The top 15 authors with the highest number of publications in metabolic endotoxemia and gut microbiota.

**Rank**	**Author**	**Country**	**Publications**	**% Of (1,585)**	**Citations**	**Average citations**
1	Cani, Patrice D	Belgium	34	2.15%	19,890	585.00
2	Leelahavanichkul, Asada	Thailand	20	1.26%	580	29.00
3	Delzenne, Nathalie M	Belgium	19	1.20%	12,175	640.79
4	Bajaj, Jasmohan S	USA	14	0.88%	1,716	122.57
5	Burcelin, Remy	France	14	0.88%	9,670	690.71
6	Violi, Francesco	Italy	13	0.82%	795	61.15
7	Cammisotto, Vittoria	Italy	11	0.69%	785	71.36
8	Everard, Amandine	Belgium	11	0.69%	6,523	593.00
9	Zhao, Liping	China	11	0.69%	3,083	280.27
10	Carnevale, Roberto	Italy	10	0.63%	754	75.40
11	Gasbarrini, Antonio	Italy	10	0.63%	567	56.70
12	Keshavarzian, Ali	USA	10	0.63%	2,544	254.40
13	Saisorn, Wilasinee	Thailand	10	0.63%	295	29.50
14	Torres, Nimbe	Mexico	10	0.63%	457	45.70
15	Tovar, Armando R	Mexico	10	0.63%	457	45.70

The information and detailed profiles of the top 10 most cited authors are systematically compiled in Table 6. Among these highly cited researchers, Cani, PD (Belgium), Turnbaugh, PJ (USA), and Ley, RE (USA) secured the top three positions based on total citation counts. Notably, while Turnbaugh, PJ maintained his prominence in cumulative citations, he also emerged as the leader in average citations per publication, underscoring the broad academic impact and sustained influence of his scholarly contributions within the scientific community. The CiteSpace visualization portrays an intricate network of cited authors (Figure 6B).

**Table 6:** The top 10 cited authors with the highest number of citations in metabolic endotoxemia and gut microbiota.

**Rank**	**Cited author**	**Country**	**Citations**	**Publications**	**Average citations**	**Total link strength**
1	Cani, Patrice D	Belgium	2,132	34	62.71	55,556
2	Turnbaugh, Peter J	USA	670	1	670.00	21,494
3	Ley, Ruth E	USA	519	1	519.00	16,593
4	Bäckhed, Fredrik	USA	453	7	64.71	15,269
5	Everard, Amandine	Belgium	386	11	35.09	12,555
6	Bajaj, Jasmohan S	USA	376	14	26.86	8,188
7	Qin, Jiangjiang	China	261	1	261.00	8,833
8	Amar, Jacques	France	252	5	50.40	7,668
9	Tilg, Herbert	Austria	164	1	164.00	4,293
10	Erridge, Clett	UK	162	1	162.00	4,872

### 3.6 Co-cited references

**Figure 7:**
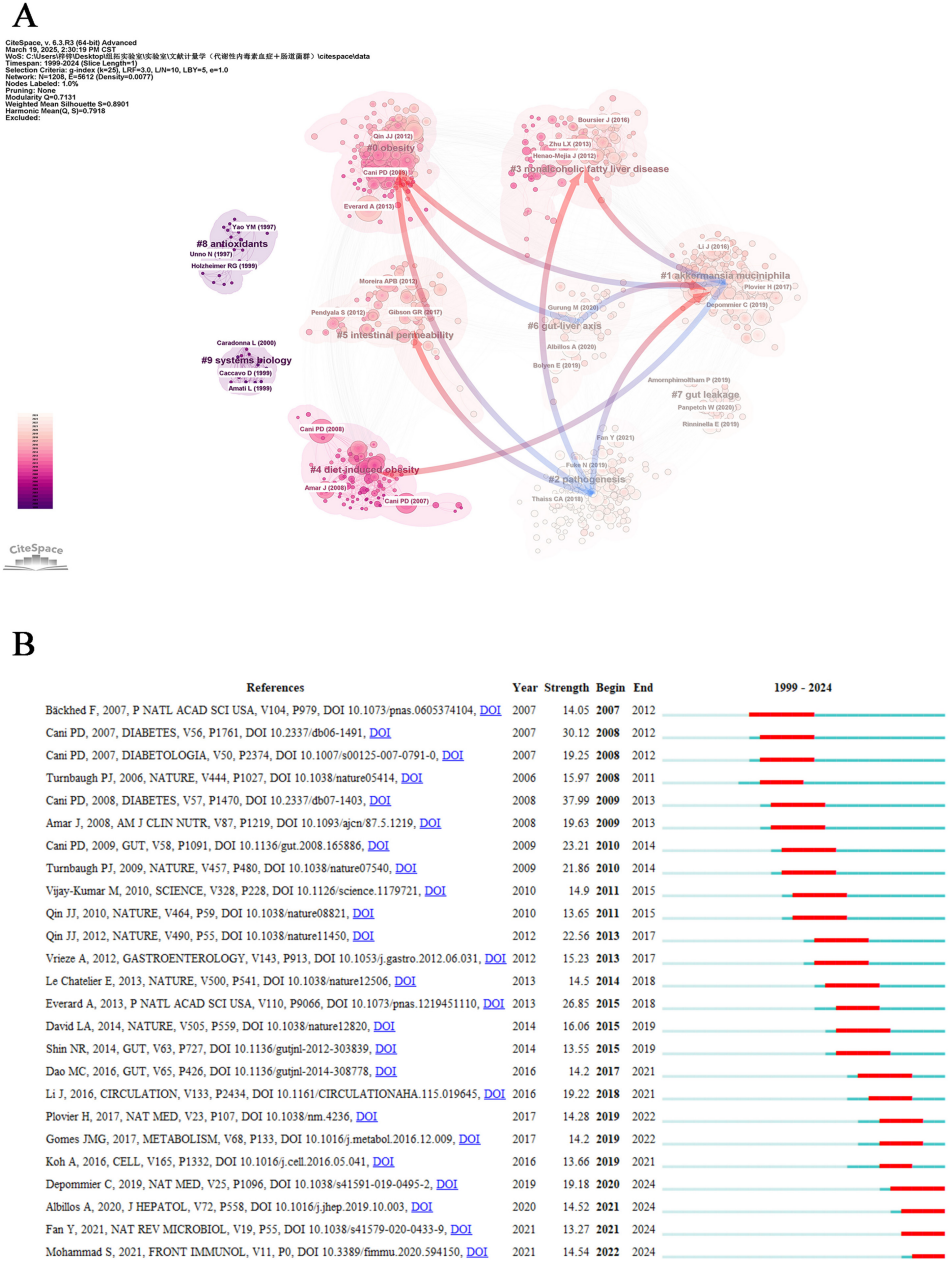
References analysis. **(A)** Cluster view of co-cited references in metabolic endotoxemia and gut microbiota. The same color represents a cluster. Pointing from one cluster to another indicates that the former cited the latter literature. **(B)** Top 25 references with the strongest citation bursts.

In bibliometric analysis, co-cited references represent foundational publications jointly cited across multiple works. Their aggregation forms knowledge clusters that reveal a discipline's research frontiers and intellectual development (Lu et al., 2020). This study employed CiteSpace software to conduct cluster analysis on retrieved literature, identifying ten distinct clusters with notable characteristics (Figure 7A). The cluster visualization demonstrated robust modular organization (modularity Q = 0.7131) and high clustering validity (mean silhouette S = 0.7918), indicating strong intra-cluster associations. Regarding cluster magnitude, “obesity” (#0) constituted the largest research domain, followed sequentially by “*akkermansia muciniphila*” (#1), “pathogenesis” (#2), “nonalcoholic fatty liver disease” (#3), and “diet-induced obesity” (#4) as core thematic areas. Trend analysis revealed that “*akkermansia muciniphila*” (#1), “pathogenesis” (#2), “gut-liver axis” (#6), and “gut leakage” (#7) have emerged as prominent research foci in recent years. Notably, “intestinal permeability” (#5) has garnered sustained academic attention as an emerging research priority. Temporal evolution analysis demonstrated persistent research interest in “obesity” (#0) and “diet-induced obesity” (#4), while interdisciplinary themes including “*akkermansia muciniphila*” (#1), “nonalcoholic fatty liver disease” (#3), “antioxidants” (#8), and “systems biology” (#9) maintained substantial scholarly engagement. Furthermore, significant interactions were identified among the clusters. “obesity” (#0) regulates “*akkermansia muciniphila*” (#1), “pathogenesis” (#2), and the “gut-liver axis” (#6), while “*akkermansia muciniphila*” (#1) concurrently influences both “pathogenesis” (#2) and the “gut-liver axis” (#6). Crucially, “nonalcoholic fatty liver disease” (#3) exerts dual effects on “*akkermansia muciniphila*” (#1) and “pathogenesis” (#2), whereas “diet-induced obesity” (#4) specifically modulates the abundance of “*akkermansia muciniphila*” (#1). Remarkably, “intestinal permeability” (#3) participates in the regulatory network of “pathogenesis” (#2) through microbial community restructuring.

Co-cited references provide foundational insights for mapping the intellectual landscape of a research domain (Chen, 2005). To determine the most impactful academic works in recent years, we performed an extensive evaluation of the 25 most citation-explosive references, which revealed prominent scholarly influence through citation surge patterns (Figure 7B). Each publication year denotes the original research timeline, while burst strength measures the intensity of scholarly attention. The temporal span (start and end years) indicates the period of sustained citation frequency, which is visualized as prominent red segments. The trio of paramount literature selections renowned for their capacity to incite explosive intensity encompasses Cani PD (2008; strength: 37.99, 2009–2013), Cani PD (2007; strength: 30.12, 2008–2012), and Everard A (2013; strength: 26.85 2015–2018), which underscore their indelible contribution to the domain of metabolic endotoxemia and gut microbiota research. Notable recent works with active citation bursts extending to our 2024 cutoff include: Depommier C (2019; strength: 19.18, 2020–2024), Albillos A (2020; strength: 14.52, 2021–2024), Fan Y (2021; strength: 13.27, 2021–2024) and Mohammad S (2021; strength: 14.54, 2022–2024) along with numerous other notable contributions that have shaped current scholarly discourse.

### 3.7 Keywords

**Figure 8:**
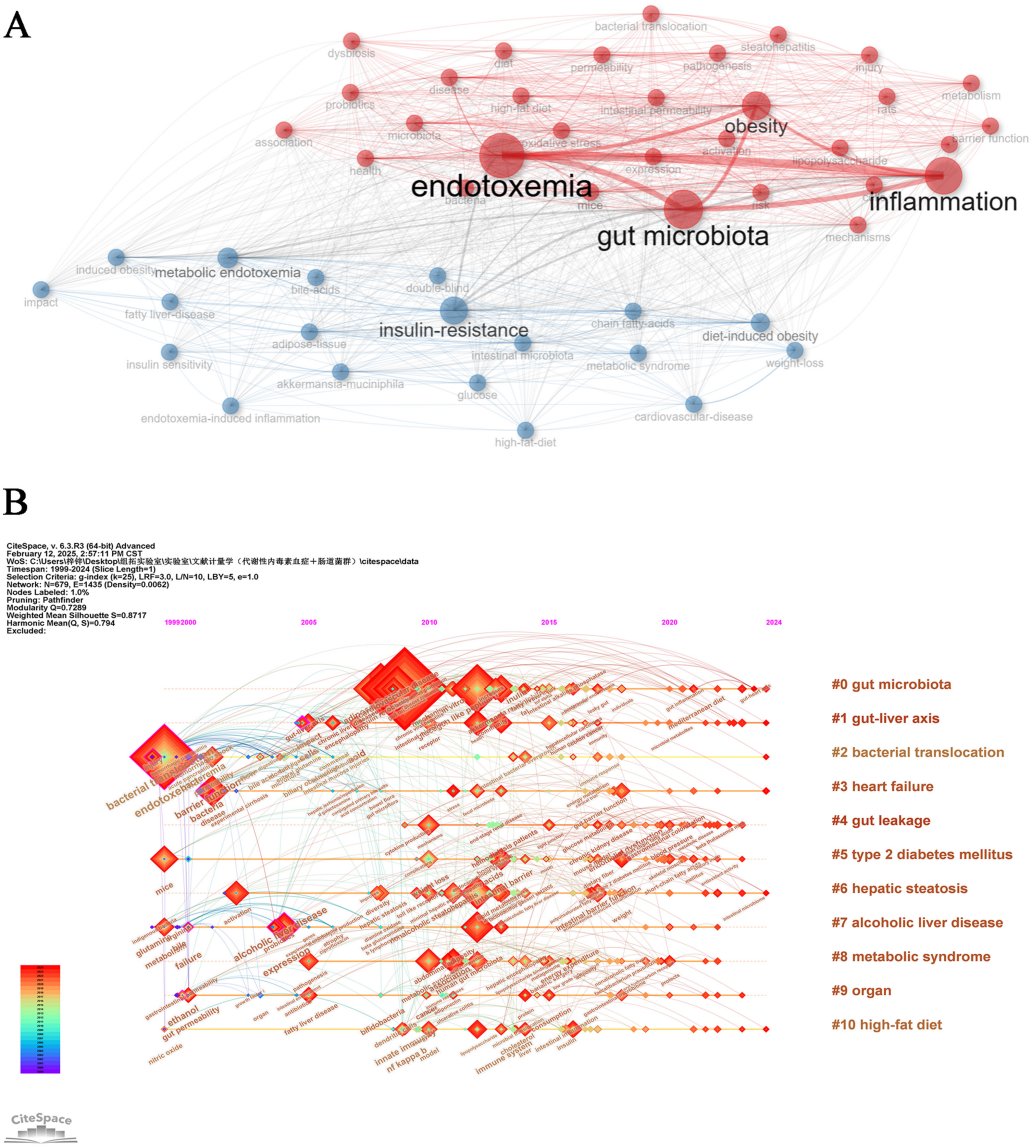
Keywords analysis. **(A)** Keywords co-occurrence Network. **(B)** Temporal overlapping co-occurrence analysis network for keywords, which enhances literature clustering through chronological expansion and identifies the initial appearance of each key term.

Keyword co-occurrence network analysis effectively elucidates research hotspots and interconnected themes in the field of metabolic endotoxemia and gut microbiota studies. Through systematic examination of distribution patterns and weighted relationships among literature keywords, this methodology delineates the knowledge architecture and central components characterizing the research domain. The analytical approach visually maps intrinsic connections between distinct research themes, establishing a robust framework for comprehending scientific inquiries within this discipline while providing a visual analytical pathway for domain exploration (Radhakrishnan et al., 2017). As illustrated in Figure 8A, the knowledge graph strategically positions “gut microbiota” and “endotoxemia” as central conceptual nodes, emphasizing their critical interrelationship in metabolic endotoxemia and gut microbiota research. This comprehensive visualization systematically integrates fundamental metrics including inflammation, insulin-resistance, and obesity while incorporating multidimensional health status assessments. The resultant analytical framework enables scholars to examine academic literature through multiple converging lenses. The implemented thematic keyword clustering strategy achieves dual objectives: maintaining precise research focus through core term selection while expanding academic inquiry boundaries via cross-keyword integration. This methodological approach significantly enhances systematic exploration of knowledge networks within the discipline, facilitating comprehensive domain understanding through strategic information architecture.

Through chronological keyword timeline mapping analysis via CiteSpace, this study systematically delineates the evolutionary trajectory of research hotspots in metabolic endotoxemia and gut microbiota from 1999 to 2024 (Figure 8B). Our current research framework integrates multiple investigative dimensions including gut microbiota (# 0), gut-liver axis (# 1), bacterial translocation (# 3), gut leakage (# 4), and metabolic syndrome (# 8). The horizontal extension of research themes along the temporal axis demonstrates both the lifecycle evolution of academic foci and reflects the field's progression from singular mechanism exploration to systemic health intervention strategies. This analytical approach effectively captures the evolution of the discipline from its early reductionist methodologies to more contemporary integrative frameworks that bridge fundamental scientific research with translational clinical applications. The observed thematic diversification underscores the maturation of gut microbiota research into a complex network of interrelated biological pathways and therapeutic targets.

### 3.8 Hotspots and trends in research

**Figure 9:**
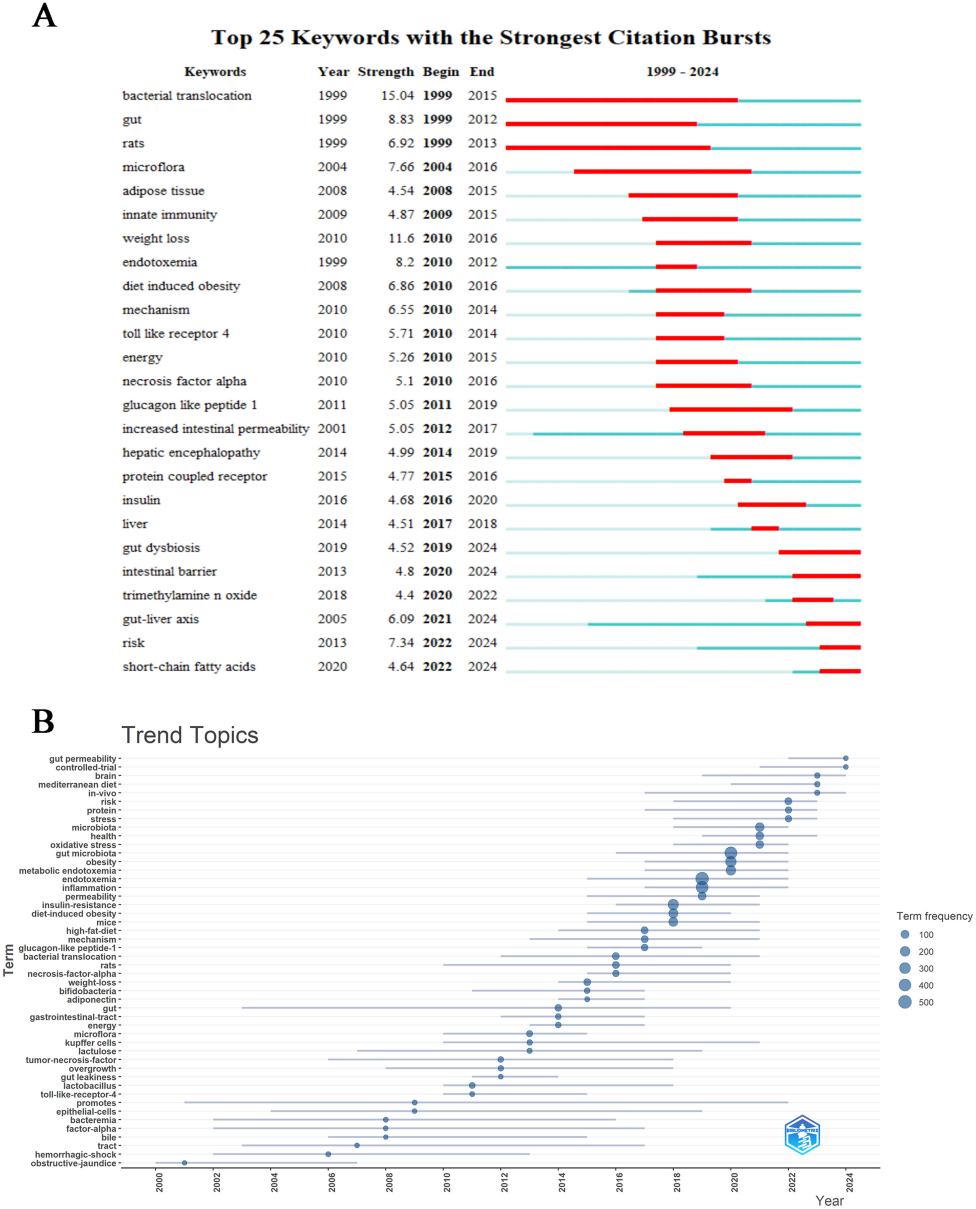
Hot topics and trends. **(A)** Trend of Topics over time. **(B)** Network map of keywords on burnout among metabolic endotoxemia and gut microbiota.

Through visual mapping analysis of research data, we systematically elucidated the academic evolutionary trends and cutting-edge hotspots within this field. The centrality metrics accurately delineate the hierarchical influence of research themes, while density indicators effectively reveal the developmental maturity of research trajectories. This cross-dimensional integration of dual metrics establishes a comprehensive framework for understanding disciplinary dynamics (Hernández-González et al., 2023). As illustrated in Figure 9A, CiteSpace's robust analytical capabilities have enabled precise detection of emerging research directions within the discipline, specifically identifying 25 core keywords demonstrating pronounced growth trajectories. The analysis reveals sustained academic activity across multiple interdisciplinary themes in recent years, including “short-chain fatty acids (2022–2024),” “risk (2022–2024),” “gut-liver axis (2021–2024),” “intestinal barrier (2020–2024)” and “gut dysbiosis (2020–2024).” The temporal distribution patterns and intensity metrics of these high-frequency burst terms suggest that these research fronts not only represent current scientific priorities but are also projected to maintain their academic prominence in the foreseeable future, indicating enduring research value and developmental potential. The keyword trend analysis depicted in Figure 9B presents a compelling graphical representation of evolving research priorities across decades. From 2000 to 2024, scientific investigations predominantly focused on three core themes: “endotoxemia,” “gut microbiota,” and “inflammation,” which emerged as persistent research anchors. Concurrently, secondary themes including “obesity,” “insulin resistance,” and “metabolic endotoxemia” maintained consistent academic attention. Analysis revealed “promotes” maintained the longest temporal span of relevance (2001–2022), underscoring its pivotal role in conceptual advancements within this domain. Post-2022 observations identified paradigm shifts with emerging terminologies such as “risk,” “protein,” “stress,” “brain,” “Mediterranean diet,” and “*in vivo*” gaining prominence in scholarly discourse. Notably, “gut permeability” and “controlled-trial” emerged as the most prominent keywords in 2024, potentially indicating new frontiers in contemporary research on metabolic endotoxemia and intestinal microbiome interactions.

## 4 Discussion

### 4.1 Analysis of the geographical distribution of publications

The global academic landscape demonstrates marked regional and structural disparities in research productivity and influence. China dominates quantitative output with 26.88% of total publications, while USA maintains qualitative leadership through its highest H-index (93) and total citations (32,688). France distinguishes itself with exceptional research depth, evidenced by a remarkable average citation rate of 183.42. International collaboration networks remain heavily concentrated among developed nations, with institutional hubs like the University of California system (USA, BC 0.17) and Inserm (France, 0.12) serving as critical nexus points. In contrast, institutions from developing countries, such as Thailand's Chulalongkorn University, exhibit near-zero intermediary centrality, underscoring persistent North-South collaboration divides. At the individual level, scholars like Belgium's Cani PD exemplify high-impact research excellence, though most prolific authors demonstrate significant quality disparities and operate within fragmented collaboration networks, as reflected in the uniformly low intermediary centrality observed among top researchers. A pivotal contribution to the field emerged from Cani PD's seminal 2008 publication (Strength 37.99) (Figure 7B), which first established the mechanistic link between gut microbiota alterations and metabolic endotoxemia through intestinal permeability modulation (Cani et al., 2008). This foundational work continues to shape contemporary investigations into microbiome-mediated metabolic disorders. Strategic recommendations emphasize the need for cross-regional collaboration frameworks leveraging existing hub institutions, targeted enhancement of intermediary capacities in developing country institutions, and the formation of interdisciplinary consortia integrating high-impact scholars. Such coordinated efforts could address systemic imbalances between quantity and quality while mitigating core-periphery structural tensions, ultimately fostering more equitable global scientific ecosystems.

It is important to highlight that while China has achieved a leading position in the volume of publications, there remains a notable absence of prominent authors and institutions in this field. To address this, China should transition from a “scale-driven” approach to a dual emphasis on “quality and network.” By enhancing international core collaborations (such as integrating into the hub networks led by USA and Europe), nurturing top-tier research teams (focusing on high citation rates for individual papers), and refining evaluation mechanisms (emphasizing originality and impact), China can gradually bridge the gap between its quantitative advantages and qualitative strength. This strategic shift will facilitate an upgrade from being an “academic power” to becoming a leader in academic excellence.

### 4.2 Analysis of the most influential journals and co-cited references

Internationally renowned journals, such as Nutrients, International Journal of Molecular Sciences, and PLOS One, have shown special receptivity for studies related to metabolic endotoxemia and gut microbiota. Researchers are advised to routinely monitor these active publication platforms to maintain awareness of emerging scientific developments. Furthermore, investigations into metabolic endotoxemia and gut microbiota inherently exhibit a multidisciplinary and integrative nature (Figure 5), primarily drawing upon foundational knowledge from disciplines including molecular, biology, immunology, medicine, medical, clinical, physics, materials, chemistry, ecology, earth, marine. This interdisciplinary convergence underscores the complexity of biological interactions within metabolic-endotoxemia-related pathophysiology and microbial community dynamics.

The literature co-citation clustering analysis has significantly advanced our understanding of metabolic endotoxemia in relation to gut microbiota, particularly by elucidating the mechanisms through which intestinal microbial communities influence systemic inflammatory responses. Notably, analysis of the top 25 most-cited references reveals emerging trends in recent scholarship, suggesting novel directions for investigating the bidirectional relationship between metabolic endotoxemia and gut microbiome dysbiosis. Recent landmark studies have contributed substantially to this evolving paradigm. Depommier et al. conducted a single-center intervention trial demonstrating that supplementation with *Akkermansia muciniphila* effectively reduced pro-inflammatory blood biomarkers without inducing significant alterations in gut microbiota composition, highlighting its potential as a therapeutic agent for metabolic inflammation (Depommier et al., 2019). Concurrently, Albillos et al. proposed the gut-liver axis as a critical regulatory interface, functioning to restrict systemic dissemination of microbial products and toxins (Albillos et al., 2020). Their work particularly emphasizes how alcohol consumption disrupts this axis through multiple interconnected pathways, simultaneously enhancing microbial translocation and creating a pro-inflammatory hepatic microenvironment. From a translational perspective, Fan and Pedersen (2021) systematically reviewed current knowledge regarding microbial-derived metabolites and their interactions with host metabolism, identifying both physiological homeostasis and pathogenesis of metabolic disorders. Their synthesis emphasizes microbiota-targeted interventions for metabolic optimization, while outlining promising directions for future basic and clinical research in microbial therapeutics. Complementing these findings, Mohammad and Thiemermann (2020) examined pharmacological and dietary strategies for mitigating chronic low-grade inflammation associated with endotoxemia, proposing actionable clinical approaches informed by microbiome modulation. This collective body of research underscores the significance of gut microbiota modulation in managing metabolic endotoxemia while highlighting emerging trends in microbiome-host interaction studies. The integration of mechanistic investigations with translational interventions presents new opportunities for developing personalized therapeutic regimens targeting gut-derived inflammation.

### 4.3 Analysis of the hotspots and trends

Through bibliometric analysis utilizing CiteSpace, emerging research trends and hotspots were identified with keyword burst detection serving as a critical indicator of conceptual evolution. Figure 9A demonstrates the citation landscape through the top 25 most referenced keywords, revealing historical research priorities in the investigation of metabolic endotoxemia and gut microbiota interactions. Notably, recent years have witnessed significant surge in thematic clusters including “gut dysbiosis,” “intestinal barrier,” “gut-liver axis,” and “short-chain fatty acids,” exhibiting sustained citation bursts that suggest their continued prominence in future investigations. While the generic term “risk” displayed comparable citation activity, its exclusion from substantive discussion is warranted due to insufficient contextual specificity. The persistent academic focus on these four biologically meaningful themes highlights their potential as frontier research areas, offering both methodological and mechanistic insights into the pathophysiological interplay between host metabolism and microbial communities. This analytical framework provides a holistic perspective on current research trajectories while identifying promising directions for advancing our understanding of metabolic endotoxemia pathogenesis and microbiota-mediated regulatory pathways.

#### 4.3.1 Gut dysbiosis

The core manifestation of gut microbiota dysbiosis lies in the comprehensive disruption of microbial community architecture and functional interconnectivity. In obesity and metabolic syndrome pathogenesis, a hallmark microbial signature emerges as the elevated *Firmicutes*-to-*Bacteroidetes* (F/B) ratio, which mechanistically enhances host energy harvest efficiency through optimized nutrient processing (Turnbaugh et al., 2006). This ecological imbalance is further exacerbated by pathobionts exhibiting altered metabolic programming. Notably, Desulfovibrio species generate hydrogen sulfide (H_2_S) that epigenetically primes pro-inflammatory responses through histone deacetylase (HDAC) inhibition, establishing persistent low-grade inflammation via chromatin remodeling (Blachier et al., 2019). Concurrently, dysregulated biosynthesis of secondary bile acids, particularly deoxycholic acid, disrupts lipid homeostasis through Farnesoid X receptor (FXR)-mediated signaling cascades, ultimately contributing to hepatic insulin resistance and impaired glucose regulation (Sayin et al., 2013). These pathological metabolite fluxes collectively signify the systemic collapse of microbiota-host crosstalk, representing a critical transition from commensal metabolism to disease-promoting pathophysiology.

The disruption of intestinal barrier integrity serves as the pivotal pathological mechanism linking gut dysbiosis to metabolic endotoxemia. Experimental evidence demonstrates that coordinated downregulation of key tight junction proteins (including occludin, claudin-4, and ZO-1) coupled with diminished mucus layer thickness - mediated through transcriptional suppression of Muc2 gene expression - collectively contribute to heightened intestinal epithelial permeability (Vaishnava et al., 2011; Johansson et al., 2015). This compromised barrier function facilitates the systemic translocation of microbial-derived molecules, particularly LPS, with circulating LPS concentrations in metabolic endotoxemia patients reaching 2-3-fold higher than physiological levels (Laugerette et al., 2012). Notably, LPS initiates a pro-inflammatory cascade through TLR4/CD14-MyD88-dependent activation of NF-κB signaling, driving excessive production of cytokines such as TNF-α and IL-6 (Frantz et al., 1999). Concurrently, sustained LPS exposure activates the NLRP3 inflammasome pathway, triggering pyroptotic cell death in adipose tissue macrophages (Vandanmagsar et al., 2011). Furthermore, chronic LPS challenge induces functional impairment of hypothalamic pro-opiomelanocortin (POMC) neurons, disrupting leptin signaling transduction and creating a self-perpetuating cycle of energy homeostasis dysregulation through compromised gut-brain axis communication (Thaler et al., 2012). This pathophysiological continuum establishes a vicious feedback loop between peripheral metabolic dysfunction and central nervous system regulatory failure.

#### 4.3.2 Intestinal barrier

The intestinal barrier, as the core defense line for maintaining gut homeostasis, exhibits multidimensional protective mechanisms with intricate molecular links to the pathogenesis of metabolic diseases. The integrity of the physical barrier relies not only on classical tight junction proteins like occludin and claudins but also involves dynamic interactions between junctional adhesion molecule JAM-A and ZO-1, which regulate paracellular permeability. Dysregulated activation of the RhoA/ROCK signaling pathway induces excessive cytoskeletal contraction in epithelial cells, creating nanoscale structural defects (Odenwald and Turner, 2017). For the chemical barrier, the spatiotemporal secretion patterns of MUC2 mucin in the mucus layer are finely controlled by the Notch-ATOH1 signaling axis. Abnormal differentiation of goblet cells leads to region-specific thinning of the mucus layer, creating entry points for pathogens (Pelaseyed et al., 2014). Notably, gut microbiota-derived tryptophan metabolites (e.g., indole-3-propionic acid) enhance epithelial junctional strength via aryl hydrocarbon receptor (AhR) activation, revealing direct microbial regulation of barrier integrity (Rothhammer and Quintana, 2019). This multi-layer barrier system plays a key role in maintaining microbial spatial separation by interacting with host immune dynamic.

Barrier dysfunction triggers cascading pathological events. Gram-negative bacterial outer membrane vesicles (OMVs) deliver l LPS not only via TLR4-mediated inflammatory signaling but also through membrane fusion-dependent delivery of NLRP3 inflammasome activators, inducing mitochondrial reactive oxygen species (mtROS) bursts and sustaining proinflammatory microenvironments (Yang et al., 2020). This dual activation mechanism explains the chronic low-grade inflammation in metabolic endotoxemia. High-fat diet-induced intestinal hypoxia suppresses butyrate-producing bacteria (e.g., *Faecalibacterium prausnitzii*), reducing butyrate levels below the threshold required for HDAC inhibition, thereby promoting proinflammatory gene expression (Chang et al., 2014).

#### 4.3.3 Gut-liver axis

The gut-liver axis, a paradigm of multi-organ crosstalk, has witnessed significant breakthroughs in molecular mechanism elucidation. Gut microbiota-derived secondary bile acids (e.g., deoxycholic acid) regulate hepatic glucose/lipid metabolism through nuclear receptor FXR activation (Yang et al., 2021) while concurrently modulating hepatic inflammatory responses via TGR5-mediated interorgan signaling (Thibaut and Bindels, 2022). Notably, microbial dysbiosis-induced tryptophan metabolic disturbances reduce indole derivatives (e.g., indolepropionic acid), which serve as endogenous ligands for AhR essential for maintaining intestinal barrier integrity through tight junction protein (occludin, claudin-5) regulation (Rothhammer and Quintana, 2019). Subsequent barrier dysfunction exposes the liver to dual insults: endotoxin assault and persistent gut-derived pathogen-associated molecular patterns, mechanistically explaining the characteristic TLR4/MyD88 pathway hyperactivation in non-alcoholic steatohepatitis (NASH) patients (Si et al., 2022). Emerging evidence reveals a reciprocal regulatory loop where hepatic stellate cell-derived fibroblast growth factor 19 (FGF19) modulates intestinal bile acid reabsorption efficiency (Kir et al., 2011).

Metabolic endotoxemia is characterized by abnormally elevated levels of circulating LPS, which forms a vicious cycle with gut-liver axis dysfunction. High-fat diets or intestinal dysbiosis can disrupt the intestinal mucosal barrier through multiple mechanisms: on the one hand, excessive saturated fatty acids in the diet (such as palmitic acid) can directly inhibit the expression of tight junction proteins like occludin and ZO-1 in intestinal epithelial cells, leading to “leakiness” of the intestinal wall; on the other hand, proliferation of specific pathogenic bacteria (e.g., *Enterobacteriaceae*) secretes proteases that degrade intercellular adhesion molecules, further increasing intestinal permeability. This dual insult makes it easier for endogenous LPS and bacterial fragments within the intestine to enter the liver via the portal vein. The Kupffer cells in the liver, serving as the core organ for metabolic detoxification, recognize LPS through TLR4, activating NF-κB and NLRP3 inflammasome pathways. This not only drives the sustained release of pro-inflammatory cytokines (such as IL-1β and TNF-α) but also disrupts insulin signaling through JNK/IRS-1 phosphorylation pathways, forming the pathological basis for chronic low-grade inflammation and insulin resistance (Hersoug et al., 2016). Recent research has revealed that intestinal microbial metabolites play a pivotal role in regulating this process. For instance, probiotics such as bifidobacteria activate GPR43 receptors in intestinal epithelia through short-chain fatty acids (SCFAs), enhancing tight junction protein assembly while inhibiting HDAC to maintain intestinal immune homeostasis (Corrêa-Oliveira et al., 2016). Notably, bile acid metabolism exhibits bidirectional regulatory characteristics in this context: activation of FXR inhibits the expression of LPS synthesis genes (e.g., those related to lipid A biosynthesis) in the intestinal microbiota, whereas G protein-coupled bile acid receptor (TGR5) improves glucose metabolism by inducing glucagon-like peptide-1 (GLP-1) secretion from enteroendocrine cells (Duboc et al., 2014). Animal experiments have shown that combined use of FXR agonist obeticholic acid and specific probiotic strains (e.g., *Bifidobacterium animalis subsp. lactis* LKM512) synergistically reduces plasma LPS levels and restores hepatic insulin sensitivity (Suzuki et al., 2022). Current research frontiers are focusing on developing precise intervention strategies targeting the gut-liver axis. Metagenomic analysis has found a significant negative correlation between the relative abundance of butyrate-producing bacteria (e.g., *Faecalibacterium prausnitzii*) and serum LPS-binding protein (LBP) concentration, suggesting potential therapeutic benefits of specific strain transplantation (Sokol et al., 2017). Furthermore, recent advances in engineered bacteria technology have made it possible to modify Escherichia coli to express alkaline phosphatase, providing a novel biological therapy for metabolic endotoxemia by effectively degrading free LPS in the intestine through synthetic biology approaches (Kurtz et al., 2019). These groundbreaking discoveries not only deepen our understanding of the mechanisms underlying metabolic endotoxemia but also lay a theoretical foundation for the development of multi-target combination therapies.

#### 4.3.4 Short-chain fatty acids

SCFAs, as the core metabolites of dietary fiber metabolism by intestinal microbiota, exhibit biological functions that extend beyond mere nutritional provision to include epigenetic regulation and fine-tuning of systemic immune homeostasis. Although acetic acid, propionic acid, and butyric acid share some metabolic pathways, their targets and effects display significant heterogeneity. Butyric acid induces chromatin relaxation by inhibiting HDAC, thereby promoting the expression of FOXP3, a key transcription factor for regulatory T cells (Treg), and enhancing intestinal immune tolerance (Atarashi et al., 2013). Additionally, as an endogenous ligand for the GPR109A receptor, butyric acid drives the polarization of colonic macrophages toward the anti-inflammatory M2 phenotype and inhibits the release of pro-inflammatory factors such as IL-1β by activating SIRT3, a negative regulator of NLRP3 inflammasome activation (Singh et al., 2014). Propionic acid, on the other hand, is preferentially taken up by the liver through portal vein circulation, activating the PPARγ signaling pathway to inhibit the expression of fatty acid synthase (FASN) while upregulating adiponectin secretion; this dual mechanism offers unique advantages in improving insulin sensitivity (Chambers et al., 2015). Notably, the concentration gradient effect of SCFAs reveals their functional complexity: physiological concentrations (1–5 mM) of butyric acid promote intestinal epithelial cell proliferation via the Wnt/β-catenin pathway, whereas high concentrations (>10 mM) trigger apoptosis by inducing p21 and Bax expression; this biphasic effect may be associated with mitochondrial metabolic reprogramming (Donohoe et al., 2011).

In the pathological context of metabolic endotoxemia, the regulation of LPS translocation by SCFAs exhibits spatio-temporal specificity and receptor dependence. Local butyric acid in the intestine enhances mitochondrial oxidative phosphorylation by activating the AMPK/PGC-1α axis, promoting O-GlcNAc glycosylation of the tight junction protein occludin, and thus strengthening the mechanical stability of the intestinal epithelial barrier (Peng et al., 2009). Systemically circulating propionic acid blocks LPS-induced MyD88-IRAK4 signaling cascades by inhibiting the endocytosis of the TLR4/MD2 complex in monocytes, a process involving SCFAs' modulation of cholesterol content in membrane lipid rafts (Haghikia et al., 2022). It is noteworthy that decreased abundance of butyrate-producing bacteria (e.g., *Faecalibacterium prausnitzii*) can lead to reduced intestinal sIgA secretion, making it easier for LPS to penetrate the mucus layer, a phenomenon particularly prominent in obese individuals (Sokol et al., 2008). Furthermore, SCFAs inhibit the ability of NF-κB to bind to promoter regions of target genes through acetylation of histone H3K9, thereby reducing the transcriptional activity of TNF-α and IL-6 (Vinolo et al., 2011). However, this anti-inflammatory effect may be offset by HDAC3 overexpression induced by a high-fat diet (Krautkramer et al., 2016). Future research should integrate metabolomics, epigenomics, and microbiomics data to elucidate the precise regulatory networks of SCFAs in individualized nutritional interventions.

The pathogenesis of metabolic endotoxemia primarily stems from the synergistic interaction between gut microbiota dysbiosis and intestinal barrier dysfunction, with pathological effects being amplified through the gut-liver axis. The core mechanism by which gut dysbiosis drives endotoxemia involves compositional shifts favoring Gram-negative bacteria (particularly *Enterobacteriaceae*), leading to increased production and release of LPS (Cani et al., 2008; Le Chatelier et al., 2013). Concurrently, intestinal barrier dysfunction manifests as comprehensive impairment across its three protective layers: (1) physical barrier disruption (downregulation of tight junction proteins including occludin and ZO-1), (2) chemical barrier deficiency (thinned mucus layer and reduced antimicrobial peptides), and (3) immunological barrier compromise (diminished sIgA secretion and immune cell dysregulation). These impairments collectively facilitate LPS translocation into the portal circulation (Bischoff et al., 2014; Chelakkot et al., 2018). Although overlapping mechanisms exist (e.g., microbial dysbiosis can directly damage barrier integrity), their pathological emphases differ: dysbiosis primarily enhances “toxin production,” whereas barrier dysfunction represents “defensive system failure.”

Translocated LPS activates hepatic Kupffer cells via TLR4, triggering NF-κB-mediated inflammatory pathways and subsequent overproduction of pro-inflammatory cytokines (e.g., TNF-α, IL-6, IL-1β), thereby establishing chronic low-grade inflammation. This process underscores the gut-liver axis's critical role in exacerbating endotoxemia and promoting metabolic disorders such as insulin resistance and non-alcoholic fatty liver disease (NAFLD) (Szabo, 2015; Tilg et al., 2016). Within this context, SCFAs (primarily acetate, propionate, and butyrate) exhibit complex regulatory functions. As principal metabolites of dietary fiber fermentation by gut microbiota (particularly butyrate-producing Clostridium clusters), SCFAs serve as essential guardians of intestinal barrier integrity: butyrate functions as the preferred energy source for colonic epithelial cells, promoting their proliferation and repair while directly upregulating tight junction protein expression. Additionally, SCFAs reinforce barrier function through G protein-coupled receptor activation (e.g., GPR43, GPR109A) on enteroendocrine and immune cells, enhancing mucus secretion, stimulating antimicrobial peptide production, and modulating Treg cell activity (Aguilar et al., 2016; Parada Venegas et al., 2019).

Notably, paradoxical observations exist where SCFAs-mediated barrier improvements fail to mitigate systemic inflammation, particularly in advanced metabolic disorders or specific disease models. For instance, in progressive NAFLD/NASH models, butyrate supplementation improves intestinal permeability yet shows limited efficacy in reducing systemic inflammatory markers. This discrepancy may arise from persistent hepatic inflammatory activation (e.g., sustained TLR4 signaling or pyroptosis pathway activation) and/or inadequate systemic bioavailability of SCFAs due to their short half-life and limited circulation concentrations, insufficient to suppress inflammation in peripheral tissues (Ji et al., 2020; Li et al., 2022). Furthermore, SCFAs demonstrate concentration- and cell type-dependent immunomodulatory effects, occasionally exhibiting pro-inflammatory properties under specific microenvironmental conditions (Vinolo et al., 2011). Thus, the net impact of SCFAs on endotoxemia and systemic inflammation depends on a complex equilibrium between intestinal barrier restoration, inflammatory status of metabolic organs, and SCFAs bioavailability (Ríos-Covián et al., 2016).

### 4.4 Future perspectives on metabolic endotoxemia and gut microbiota

Based on current research, future investigations in the field of metabolic endotoxemia and gut microbiota will focus on innovative integration of mechanistic exploration and precision intervention strategies. By combining multi-omics technologies (metagenomics, metabolomics, epigenomics) with artificial intelligence, researchers aim to systematically elucidate the spatiotemporal dynamics through which microbial metabolites (e.g., SCFAs, bile acids) regulate intestinal barrier integrity (tight junctions, mucus layer) and inter-organ axis networks (gut-liver, gut-brain), particularly emphasizing strain-specific functionalities (e.g., butyrate-producing gene clusters) and paradoxical phenomena (e.g., LBP's dual role, FXR's tissue-specific effects). Therapeutic innovations will evolve toward engineered and personalized approaches, including LPS-targeting synthetic microbial consortia, SCFAs delivery systems addressing concentration-dependent efficacy limitations, and combinatorial “cocktail therapies” integrating FXR agonists, HDAC inhibitors, and prebiotics. Concurrently, overcoming clinical translation challenges requires establishing multidimensional biomarker panels incorporating I-FABP, microbiota gene abundance, and inflammatory activity metrics. Patient stratification based on enterotypes and metabolic phenotypes will guide individualized interventions, with final validation through humanized models to advance the paradigm shift from disease treatment to health maintenance.

## 5 Limitation

This study acknowledges four principal limitations. First, the analysis was based on WoS database, which probably omitted the relevant publications indexed in other repositories. Second, the temporal scope of the literature review (1900–2024) excludes a small number of studies and publications predating 1900, published studies after 2024, and emerging research that has not been formally indexed yet. Third, the criteria for inclusion limited the analysis to peer-reviewed articles and reviews which means that other types of allocative publications such as conference proceedings or book chapters were excluded. Additional limitations relate to the bibliometric tools used, namely VOSviewer and CiteSpace, which are not able to perform full-text analyses and have a bias toward English-language texts. These technical limitations may introduce selection bias, which may overlook subtle contextual data and recently published works that have not been indexed or are provided in non-English formats, leading to results that are biased toward the dominance of the Western countries/China.

## 6 Conclusion

The present study was a systematic investigation of the landscape of global research on metabolic endotoxemia and gut microbiota, delineating developmental trends from 1999 to 2024. Rigorous statistical analyses of trends in publication in these fields indicate the magnitude of scholarly output during this time. China, USA and Italy were leading contributors, USA had marked academic influence and Belgian researchers dominated the top 10 most productive authors. The most important agency is undoubtedly the Inserm, the leading research organization in the country. In addition, using this analytical strategy, we distinguish evolving research themes in metabolic endotoxemia-gut microbiota research by delineating prominent knowledge frontiers. Though early studies focused on mechanisms of diet-induced obesity, more recent research has progressed to explore address pathophysiological mechanisms and systemic interactions. Most future lines of investigation should build on those basic ones.

These analytical frameworks serve as robust tools for tracking the evolution of this dynamic research domain. Visualization methodologies further identify core journals and interdisciplinary collaborations shaping the field, providing researchers with a strategic roadmap to navigate cutting-edge discoveries and advancements. Collectively, this work synthesizes the current state and intellectual dynamics of metabolic endotoxemia and gut microbiota research, establishing a critical reference framework to guide researchers in deepening their understanding of this pivotal scientific area.

## Data Availability

The raw data supporting the conclusions of this article will be made available by the authors, without undue reservation.
